# Stripline Multilayer Devices Based on Complementary Split Ring Resonators

**DOI:** 10.3390/mi13081190

**Published:** 2022-07-28

**Authors:** Eduardo Jarauta, Francisco Falcone

**Affiliations:** 1 Department of Electric, Electronic and Communication Engineering, Universidad Pública de Navarra, Campus Arrosadía, E-31006 Pamplona, Spain; jarauta.31784@e.unavarra.es; 2 Institute of Smart Cities, Universidad Pública de Navarra, Campus Arrosadía, E-31006 Pamplona, Spain; 3 Tecnologico de Monterrey, School of Engineering and Sciences, Monterrey 64849, Mexico

**Keywords:** stripline multilayer, power divider, multilayer diplexer, multilayer resonator, complementary split ring resonator

## Abstract

A new analytic design for multilayer stripline devices in planar circuit technology is presented. The Complementary Split Ring Resonator (CSRR) is used as a sub-wavelength resonant particle, which provides high-Q resonances in a compact size. The electromagnetic field distribution achieved along the stripline enables enhanced excitation of the resonators. An optimal solution for multilayer power dividers is presented, in a configuration in which each output is obtained in different layers and also in a different layer than the input line. The solution is expanded to design different devices, such as diplexers, resonators, and multi-frequency resonators, leading to vertical filters. As the resonances are achieved by stacking resonators, the effective circuit footprint is very compact. The proposed devices can be implemented in a volumetric chip fashion, allowing integration with planar transmission line circuits and flexible output connection placement. A complete analysis of the different devices is proposed, extracting and verifying their equivalent circuit models.

## 1. Introduction

The fields distribution in the stripline transmission line, building a TEM mode [1], has allowed this to be the transmission line of choice for the design of different types of devices. The fact that fields are concentrated between ground plane metallization layers provides better isolation against external fields and also avoids their own radiation effects.

The use of stripline circuits to build transitions in multi-stacked structures has also been commonly used [2,3]. In multilayer configurations, they are also used as feedline for antennas [4], structures to build multiplexers [5], multilayer couplers [6,7], circulators [8], or for completely different applications such as multi harmonic absorption circuits [9]. An analytical study of slotted stripline was proposed [10], providing insight into the first-order characterization for this type of circuit configurations.

In this work, the behavior of a stripline transmission line loaded with Split Ring Resonators (SRR) presented by Pendry [11]—along with its complementary, Complementary Split Ring Resonators (CSRR) [12]—will be proposed. SRRs take advantage of the excitation of axial magnetic fields in order to excite the main quasi-static resonance frequency, which exhibits sub-lambda characteristics (i.e., physical resonator dimensions smaller than those defined electrically by wavelengths, which inherently imply miniaturization capabilities of these structures). In the case of CSRRs, by applying Babinet’s duality principle (interchanging the role of the E and H fields and hence the metallization layers for air gap strips), excitation is achieved by axial E-field components, which are useful in the case of transmission lines such as microstrip lines. In both cases, SRRs as well as CSRRs exhibit bi-anisotropic behavior, although the main quasi-static resonances can be excited by axial H-field and axial E-field components in SRRs and CSRRs, respectively.

The integration of SRR and CSRR elements established a starting point for the miniaturization of microwave devices. They have been intensively used for all kinds of devices—from filters [13,14] to couplers [15,16]—widely used in antenna design [17,18], and in the last years, there has been a growing interest in sensors [19,20] due to the high-quality factor in the quasi static resonance [21]. The first approach of an equivalent circuit of SRR and CSRR was characterized by [22]. This approach was enhanced in different configurations [23,24,25] and for different applications [26]. In this work, the aforementioned designs are used as a starting point to analyze the structures with the enhanced circuits proposed.

Most of the designs previously described integrate CSRR or other types of resonant elements embedded within the transmission line topology, taking advantage of the EM field configuration in order to adequately excite—usually, the quasi-static resonance. In order to achieve high frequency selectivity, several resonant elements are employed, usually in a series-cascaded configuration, which increases the circuit length. Moreover, the input and output ports are given by the transmission line routing, which can be limited by factors such as bending ratios in meandered configurations, impedance mismatches, or radiation leakage effects. The proposed circuit topologies in this work take advantage of multi-stack configurations, which have the capability of reducing the circuit footprint whilst maintaining compatibility with planar transmission line ports. An additional feature of the proposed configurations is the capability of employing different facets of the volumetric multi-stack structure, which enables a higher flexibility in the input/output port location and, hence, in the integration of the devices in systems such as communication transceivers.

This work initially describes the CSRR behavior embedded in the stripline ground plane. In successive sections afterwards, different devices will be proposed, starting with power dividers and followed by the resonator, multi-frequency resonators, diplexers and filter design.

## 2. Stripline Loaded with Complementary Split Ring Resonators

**Figure 1:**
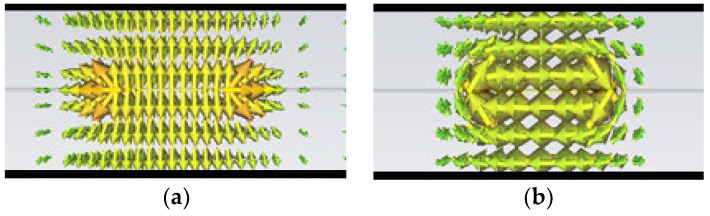
Representation of the EM field distribution in the stripline. (**a**) E-field. (**b**) H-field.

In Figure 1, the electric and magnetic fields distributions are plotted within a stripline transmission line configuration. As can be seen, a TEM mode between the input line and ground planes is excited (i.e., the E-field is axial to the ground plane). It is well known that CSRRs are excited by an electric field axial to the ring [27]. Considering this fact, the TEM mode of the stripline provides a proper excitation mode, as was already demonstrated by embedding the CSRR in the input line of a stripline in [28].

**Figure 2:**
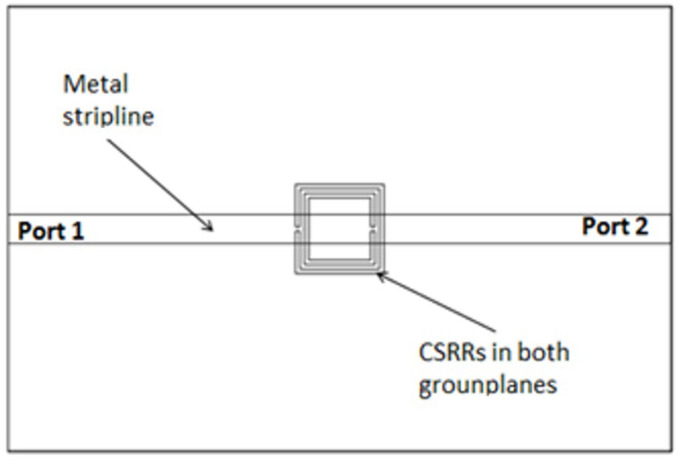
Top layout stripline loaded with SCSRR elements embedded in the top and bottom layers.

The initial step taken was to consider loading the standard stripline with CSRRs. Two identical rings are placed on the top and bottom metallization layers. In this study, square complementary Split Ring Resonators (SCSRR) will be used. The top layout is displayed in Figure 2.

**Figure 3:**
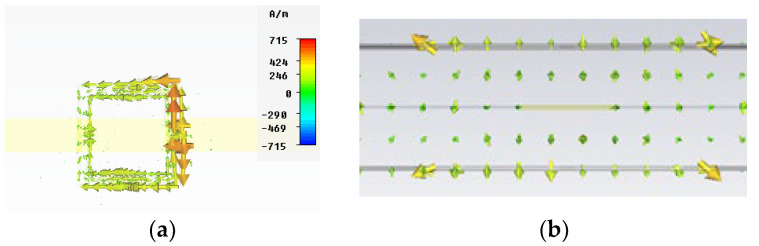
Vector surface current at a resonant frequency. (**a**) Absolute amplitude values (top view). (**b**) Vector values (front view).

The CSRR is excited in the quasi-static resonance. In Figure 3, the surface currents at the resonant frequency are plotted. The results have been obtained with the aid of the CST MW Studio transient solver, providing open boundary layers with no symmetries applied.

**Figure 4:**
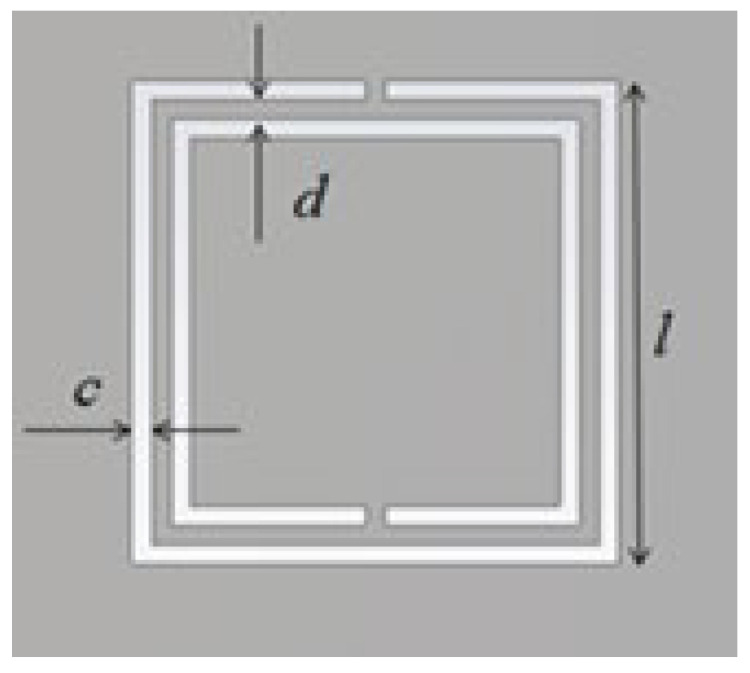
SCSRR main dimensions.

The main dimensions for the Square Complementary Split Ring Resonator used in the first part of this paper are presented in Figure 4. They have the following values: length of the square side *l* = 5 mm, thickness of the rings *c* = 0.2 mm, distance between rings *d* = 0.2 mm. These were obtained from previous quasi-static estimations derived in [22] as a function of the quasi-static resonance frequency (fixed initially, in this case, to 5.8 GHz). The substrate used for the simulation is the commercially available Rogers RO5880 with an *ɛ_r_* = 2.2 and a substrate thickness *h* = 0.79 mm.

**Figure 5:**
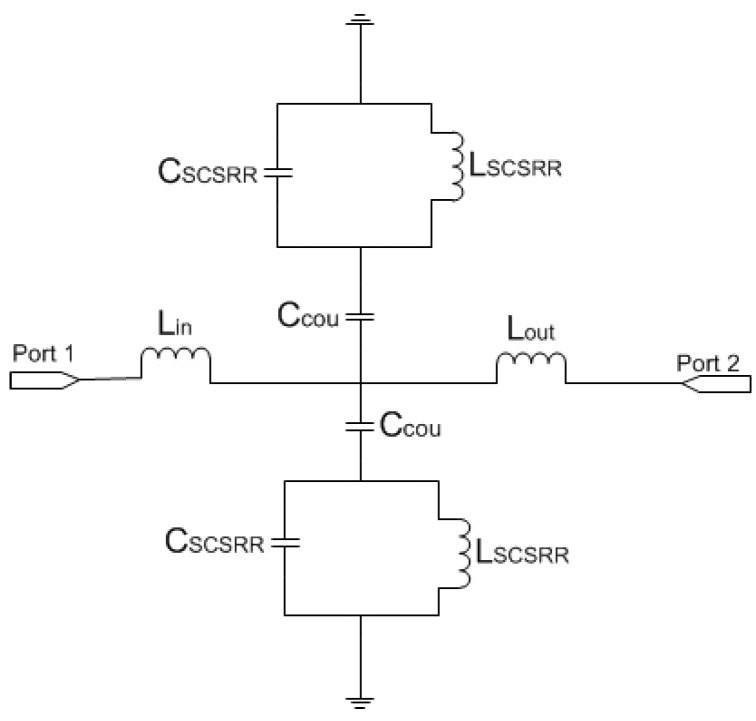
Equivalent circuit model of the stripline loaded with SCSRR.

The equivalent circuit for the SCSRR follows the same approach followed to model CSRR [21], but it is adapted to a stripline, i.e., one resonator from the feedline to each ground plane. The complete circuit is depicted in Figure 5. To calculate the values of the inductance *L_SCSRR_* and capacitance *C_SCSRR_*, the equations from [22] are used. In that research, circular CSRRs are used. To make use of those equations, the perimeter of the current flow through the SCSRR has been calculated. The stripline transmission line is characterized with the per-section inductance of the stripline line [1].

(1) $$C=\frac{\varepsilon_{r}l}{30\pi \vartheta_{0}\left(\frac{1}{\frac{w}{s}+0.041}\right)}\;\mathrm{for}\;\frac{w}{s}\;\geq \;0.35$$

(2) $$C=\frac{\varepsilon_{r}l}{30\pi \vartheta_{0}\left(\frac{1}{\frac{w}{s}-{\left(0.35-\frac{w}{s}\right)}^{2}+0.441}\right)}\;\mathrm{for}\;\frac{w}{s}<0.35$$

(3) $$L=\frac{30\pi l}{\vartheta_{0}}\;\left(\;\frac{1}{\frac{w}{s}+0.441}\right)\;\mathrm{for}\;\frac{w}{s}\;\geq \;0.35$$

(4) $$L=\frac{30\pi l}{\vartheta_{0}}\;\left(\;\frac{1}{\frac{w}{s}-{\left(0.35-\frac{w}{s}\right)}^{2}+0.441}\right)\;\mathrm{for}\;\frac{w}{s}<0.35$$

where *w* is the width of the stripline conductor and *s* is the separation between the stripline plates. In our case, *w* = 1.3 mm and *s* = 1.58 mm. That is, equations (1) and (3) will be used for the calculations. ε_r_ is the permittivity of the dielectric—in our case, ε_r_ = 2.2 and υ_0_ is the speed of light in a vacuum.

**Figure 6:**
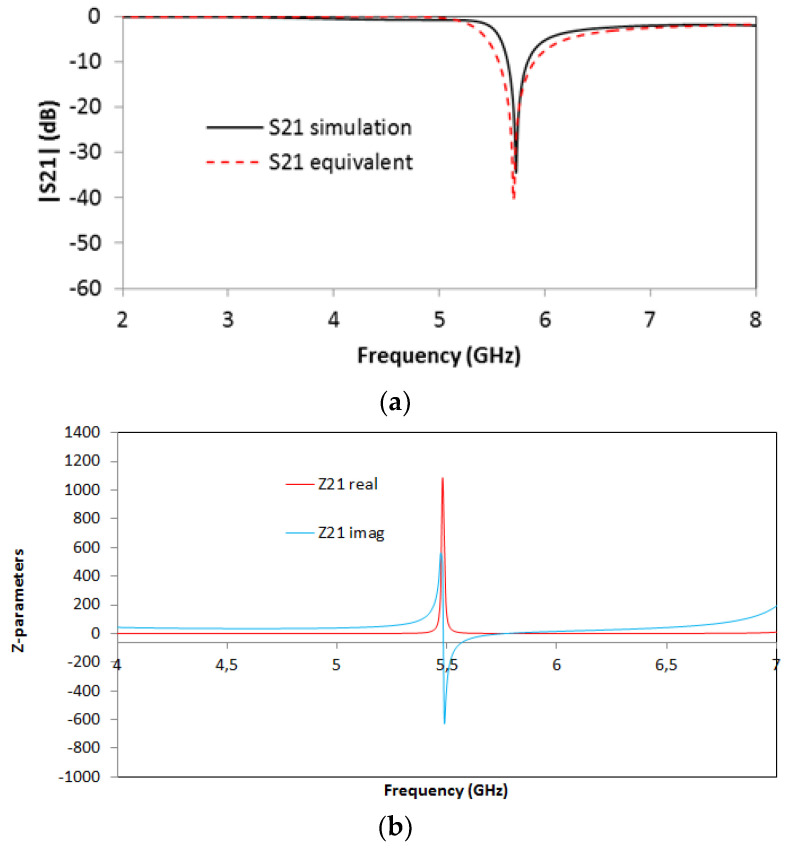
(**a**) Simulated S21 (solid black line) and simulated S21 for the equivalent circuit model (dashed red line). (**b**) Impedance graph: Z21 real part (solid red line) and Z21 imaginary part (solid blue line).

For the SCSRR’s dimensions, considering the same substrate, the following values are obtained for the components of the equivalent circuit: *L_in_* = *L_out_* = 0.995 nH. The coupler from the stripline to the resonators *C_cou_* = 0.188 pF. For the resonator values *C_SCSRR,_ L_SCSRR_*, as explained, an average of the perimeter of SCSRR is calculated. In this case, we use a length side of 4.7 mm. We obtain a perimeter of 18.8 mm. With these perimeters, we obtain the equivalent radius if the rings would be circular. In our case, *radius* = 2.992 mm. Finally, using the equations in [22] providing the radius, the separation between the rings, and the substrate dimensions, the values of the resonator are *C_SCSRR_* = 0.37 pF and *L_SCSRR_* = 1.39 nH. Figure 6 shows the simulation results in comparison with the designed circuit model.

Good agreement is achieved between the simulation and the equivalent circuit, which validates previous assumptions. A resonance at *f_0_* = 5.71 GHz is achieved, corresponding to the SCSRR resonance. There are, however, some slight differences between the results, which can be given effects such as finite circuit dimensions (which can lead to diffractive losses), non-idealities in the coupling sections (e.g., the finite length of the coupling gaps and the consideration of the edges and corners), or the effect of higher-order mode coupling in the multi-stack structure. The single resonator particle will be used in this work in order to build more complex structures. From the S-parameters, the impedance parameters are obtained. In Figure 6b, the real and imaginary parts of Z21 are represented. An abrupt change in impedance is obtained, which comes from the infinity value of the impedance and the capacitance of the resonator. It is worth noting that, in all the designs, transmission lines have been designed with a line impedance of 50 Ω, in compliance with conventional measurement port values. Given the inherent narrowband characteristics of the CSRR quasi-static resonance, impedance matching can be directly achieved, although potential requirements in line impedance could, in general, be handled by using simple λ/4 adapters or similar approaches.

## 3. Power Divider

Power dividers based on SRRs were used to design power dividers in different configurations—such as Wilkinson power dividers [29] and Bailey power dividers [30]—and also in substrate-integrated waveguide (SIW) structures [31]. The use of composite right-handed/left-handed has also been employed in the design of multilayer power dividers [32].

As explained in the introductory section, the field distribution in the stripline is homogenous, exciting a TEM mode along the transmission line. The main idea is to extract all the energy that reaches the ground plane to a new layer by using slot resonators etched on each ground plane. Coupling energy, through a slot, was successfully implemented and reported in [33]. The use of this concept to design power dividers has also been developed and reported in [34,35]. In this work, to achieve coupling to a different layer, a square complementary split ring resonator will be used. As shown in Figure 1, the E-field lines in the stripline expand from the input metallization to the ground planes. The SCSRR will be placed symmetrically on each metallization layer. The axial electric field will be used to excite each SCSRR [12]. In the schematic layout, we can see that a central metallization line acts as the input of a stripline. The ground planes of the stripline are the location for the embedded SCSRRs. These metallization layers also act as the ground plane for the microstrip line built for outputs: one on top of the device and the other one in the bottom layer.

**Figure 7:**
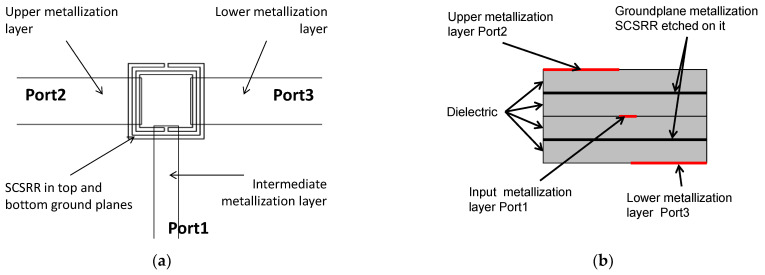
(**a**) Top view of the SCSRR within the power divider. (**b**) Front view of the device.

The layout of the proposed device is displayed in Figure 7. The main dimensions of the SCSRR are: the length of the square side *l* = 4 mm, the thickness of the rings *c* = 0.2 mm, the distance between rings *d* = 0.2 mm.

**Figure 8:**
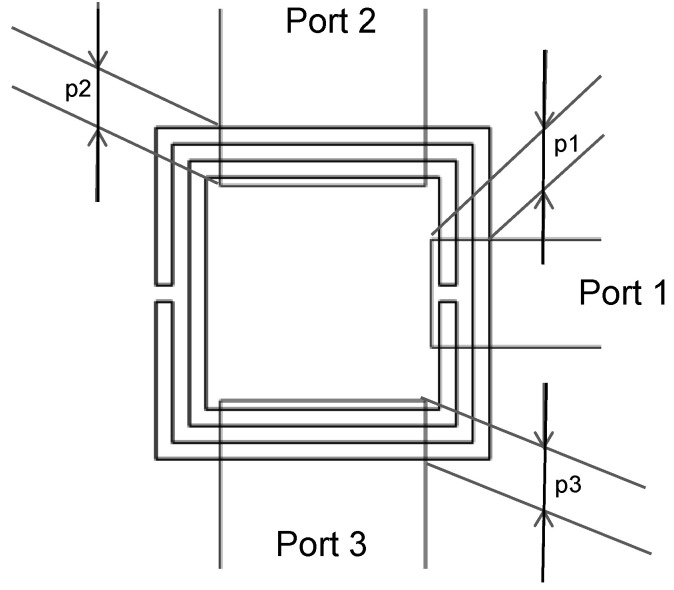
Detailed top view and relevant dimensions.

The stripline, which acts as the input, is cut as an opened stub. Both SCSRRs are placed exactly in the same position in top and bottom of this input line. The line is inserted into the rings a distance *p1*. In this design, the outputs are placed in each side of the rings without any gap. The currents flowing through the resonator will be coupled to the output lines, which are also designed as open stubs. The distances between the edge of the ring and the end of the output lines are *p2* and *p3*, respectively, as pictured in Figure 8. For the power divider, the values are *p1* = 0.7 mm, *p2* = 0.7 mm, and *p3* = 0.7 mm.

**Figure 9:**
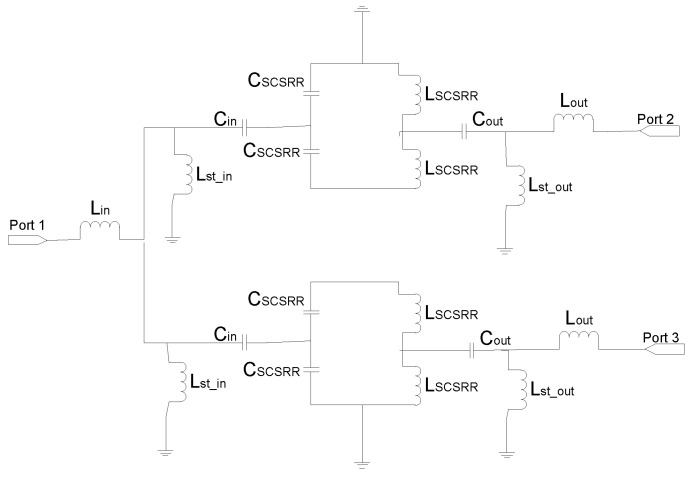
Equivalent circuit for the multi-layer power divider.

The equivalent circuit for the power divider presented above is plotted in Figure 9. The input line is characterized by the serial inductance *L_in_*. The end of the input line is represented as an open stub *L_st_in_* to the ground plane on each line. The next stage is the coupling from the input line to the resonators represented as *C_in_*. The LC components of the resonator have been split in the series couples of the coupler *C_SCSRR_* and the inductance *L_SCSRR_*. Given the circuit topology, the value of *C_SCSRR_* is double that calculated from [22], whereas the inductance *L_SCSRR_* is half of the value.

**Figure 10:**
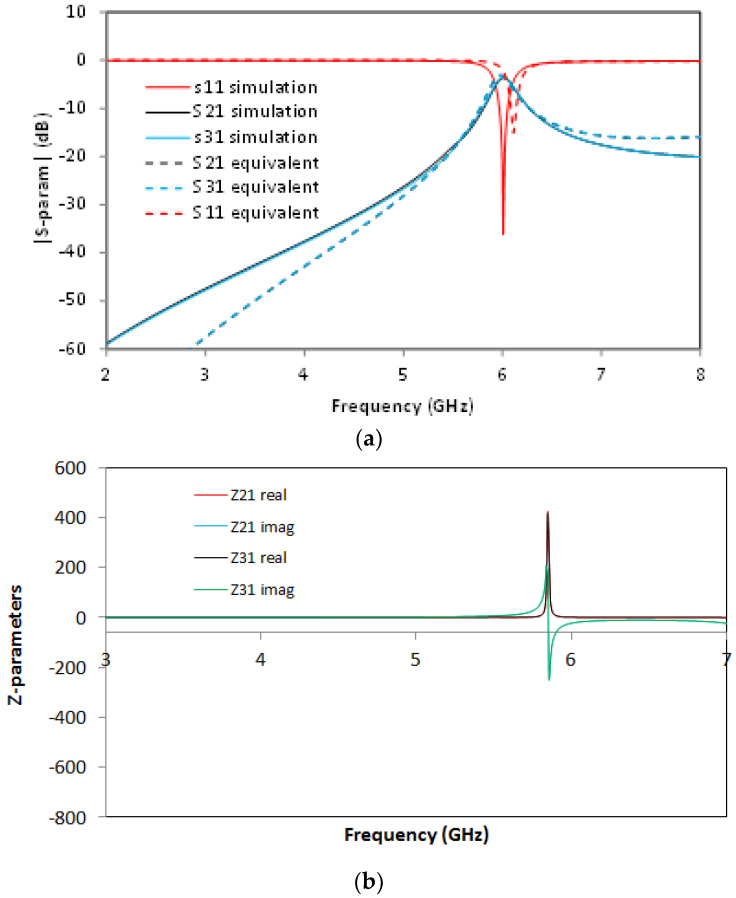
(**a**) Simulated S11 (solid red line), simulated S11 for the equivalent circuit model (dashed red line), simulated S21 (solid black line), simulated S21 for the equivalent circuit model (dashed black line), simulated S31 (solid blue line), simulated S31 for the equivalent circuit model (dashed blue line). (**b**) Impedance graph: Z21 real part (solid red line), Z21 imaginary part (solid blue line), Z31 real part (solid black line), Z31 imaginary part (solid green line).

For the SCSRR, the previously enumerated dimensions are valid, and the same substrate (Rogers RT 5880) is used. The values of the components for the resonator are calculated as explained in the previous section and following [22], with the consideration that these are square resonators already explained. For the input parameters *L_in_*, *C_in_*, *L_out_*, and *C_out_*, the capacitance and inductance per unit length is calculated. For the inductances generated as an open stub—*L_st___in_*, *L_st_out_*—an optimization method is used. Taking this into account, the calculated values for the elements in the equivalent circuit are: *L_in_* = 0.497 nH, *L_st_in_* = 1.22 nH, *C_in_* = 0.188 pF, *C_SCSRR_* = 0.74 pF, *L_SCSRR_* = 0.695 nH, *C_out_* = 0.266 pF, *L_st_out_* = 1.287 nH, *L_out_* = 0.497 nH. In Figure 10, the output response for both the simulation of the circuit and the equivalent circuit is plotted.

The output reveals an almost symmetric response to ports 2 and 3, with insertion losses S21 = −3.43 dB and S31 = −3.67 dB, respectively, which provide an efficiency of 93%. The return loss value is S11 = −36.2 dB at a central frequency *f_0_* = 6.008 GHz, and the isolation between the output ports is greater than 8 dB.

## 4. Multi-Layer Resonators

The use of resonances through holes and slots has been deeply studied. They were used to design different components such as filters [33,34], power dividers [35], couplers [36,37], or even transitions from microstrip transmission lines to waveguides [38]. In this section, the SCSRR embedded in the stripline transmission line is used to create a new device in multi-layer configuration.

**Figure 11:**
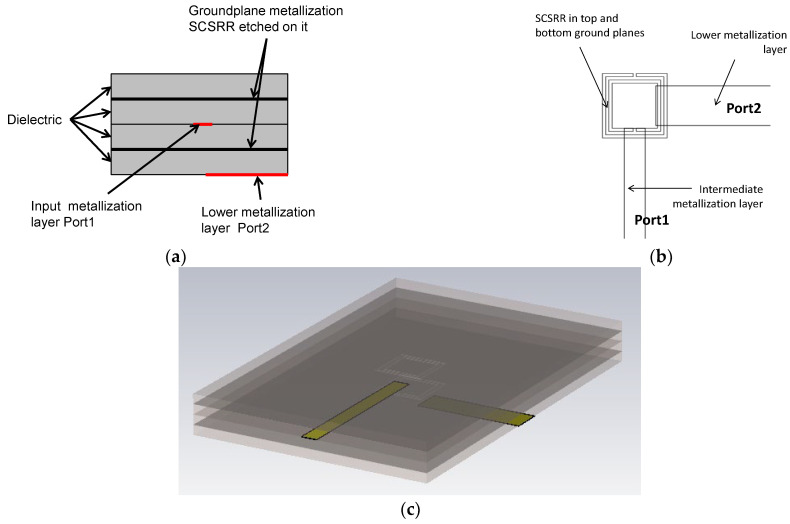
(**a**) Front view. Stacked layer distribution: (**b**) top view of the SCSRR power divider; (**c**) perspective view of the stacked layers.

The topology of this device is as follows. The input line is the metallization of a stripline. In the middle, on each ground plane, an SCSRR is embedded. Above and below the metallization layers that form the stripline, a substrate is added on each layer. The stacked distribution is shown in Figure 11a. Finally, at the bottom layer, a new metallization line is added from the center to port 2 of the circuit, building a microstrip line, as displayed in Figure 11b. To have a clear understanding of the complete structure, a perspective view is depicted in Figure 11c. The circuit has been designed in the same substrate with an ε_r_ = 2.2 and a substrate thickness *h* = 0.79 mm, with the same values of SCSRR dimensions as those in the single particle described previously.

**Figure 12:**
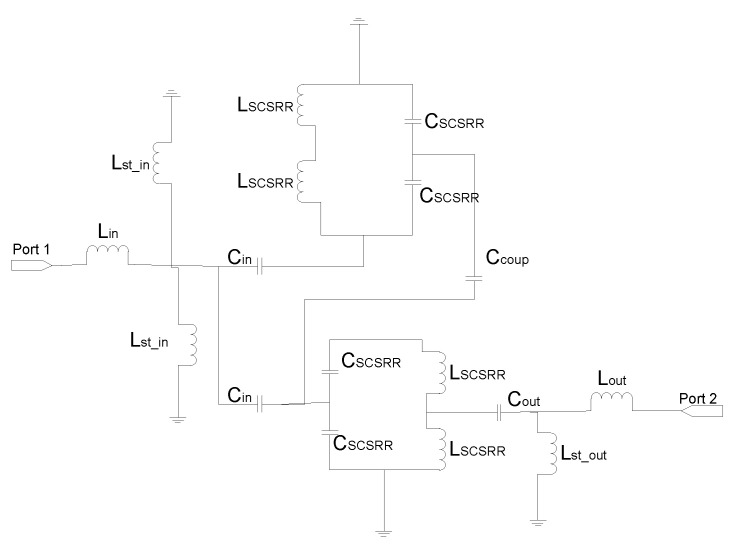
Equivalent circuit for the stripline multilayer resonator.

The equivalent circuit consists of the elements shown in Figure 12. The input stripline is modeled with an inductance per unit length *L_in_*. The input line ends over the SCSRRs. This is modeled as an open stub for each ring, *L_st_in_*. The coupling from the input line to the resonators SCSRR is modeled with capacitor *C_in_*. The resonators are characterized by their LC tank. As the capacitance and inductance are split in two serial components, the value of *L_SCSRR_* is half of the value calculated for the resonator, and the value of *C_SCSRR_* is double the value calculated for a single resonator [22]. In this device, the ring on the top ground plane is not coupled to an output line. Coupling exists between both rings, which is represented as *C_coup_*. The coupling from the ring to the output microstrip line is modeled again by a capacitor *C_out_*. As explained before, the length of the output line inserted in the SCSRR is characterized as an open stub, labeled, in this case, as *L_st_out_*. Finally, the output line is again characterized by the inductor *L_out_*.

**Figure 13:**
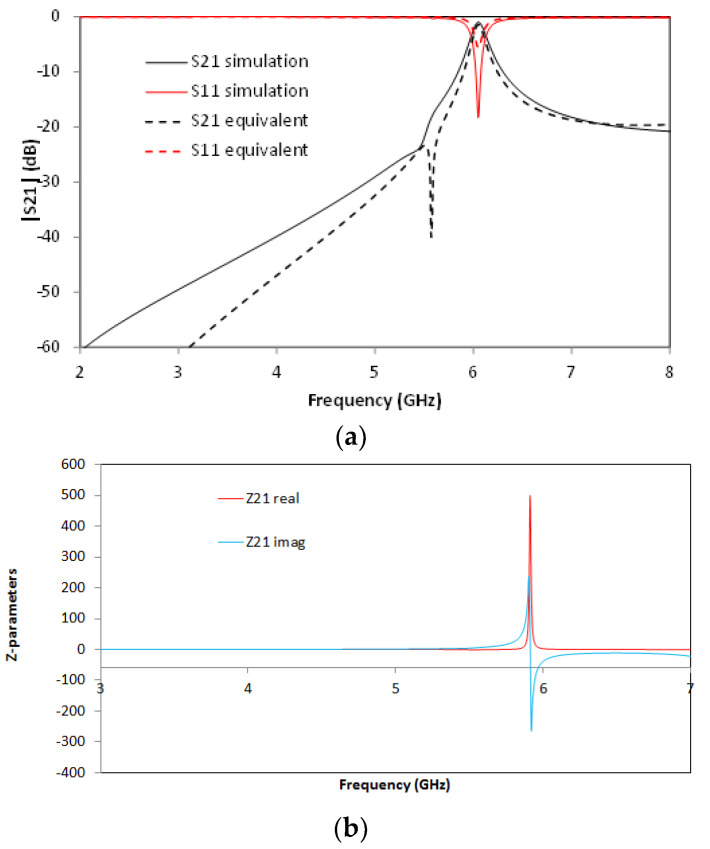
**(a**) Simulated S11 (solid red line), simulated S11 for the equivalent circuit model (dashed red line), simulated S21 (solid black line), simulated S21 for the equivalent circuit model (dashed black line). (**b**) Impedance graph: Z21 real part (solid red line), Z21 imaginary part (solid blue line).

For the ring dimensions previously stated, the values of the equivalent circuit are: *L_in_* = 0.349 nH, *L_st_in_* = 0.922 nH, *C_in_* = 0.188 pF, *L_SCSRR_* = 0.695 nH, *C_SCSRR_* = 0.740 pF, *C_coup_* = 0.039 pF, *C_out_* = 0.216 pF, *L_st_out_* = 1.210 nH, *L_out_* = 0.327 nH. In Figure 13, the simulation results are compared with the equivalent circuit model presented.

Good agreement is achieved between the circuit model presented and the results obtained in the simulation. On this device, the resonant frequency is observed at *f_0_* = 6.048 dB. The S-parameters values, at the mentioned resonant frequency, are insertion loss: S21 = −1.01 dB and return loss: S11 = −18.25 dB. The 3 dB bandwidth at the resonant frequency is 171 Mhz. With these values, the unloaded quality factor (*Q_u_*) for this resonator is calculated, giving a value of *Q_u_* = 355. As shown in [21], the resonator is excited in sub-lambda frequencies, and the dimensions of the ring are around eight times the free space wavelength at the resonance. Thus, the unloaded quality factor has a bigger value in comparison to the other resonators [39].

## 5. Multi-Frequency Resonator

**Figure 14:**
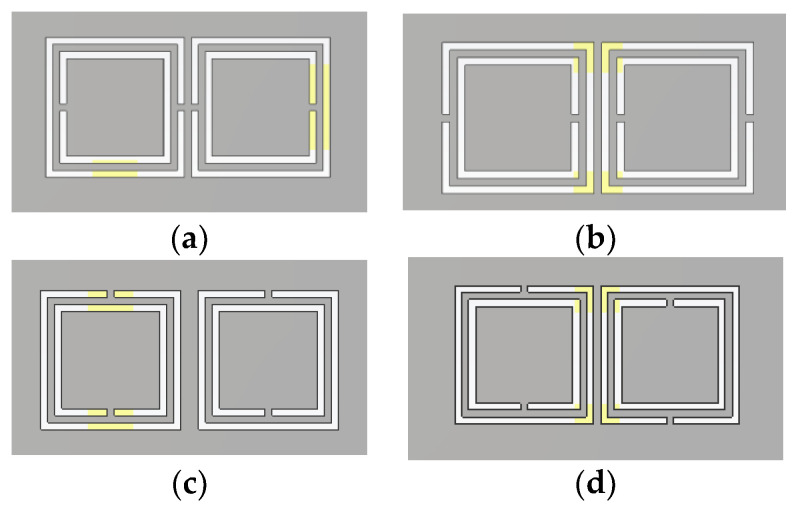
Topologies of couples of SCSRRs. (**a**) face-to-face, (**b**) back-to-back, (**c**) side-by-side, (**d**) side-by-side reversely.

Up until now, single particle devices have been used in the design of duplexers on resonators. In this section, more rings will be added in order to obtain a second resonance. Two square ring resonators were deeply studied in several combinations: face-to-face, back-to-back, side-to-side, and side reversely [39], embedded in a substrate integrated waveguide (SIW). The different combinations are presented in Figure 14.

**Figure 15:**
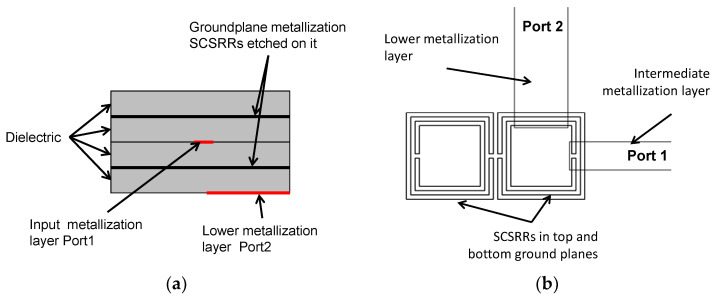
(**a**) Front view. Stacked layer distribution: (**b**) top view of the SCSRR multi-frequency resonator.

For the first device presented, two rings in the face-to-face configuration are placed on both metallization layers (Figure 15). The input line is the stripline metallization layer. The line is cut when it reaches the SCSRRs. As previously stated, a couple of SCSRRs on each layer are embedded. Finally, the output line starts perpendicular to the input line in the same SCSRR as the input but below the lower ground plane metallization layer, implementing an output microstrip line.

**Figure 16:**
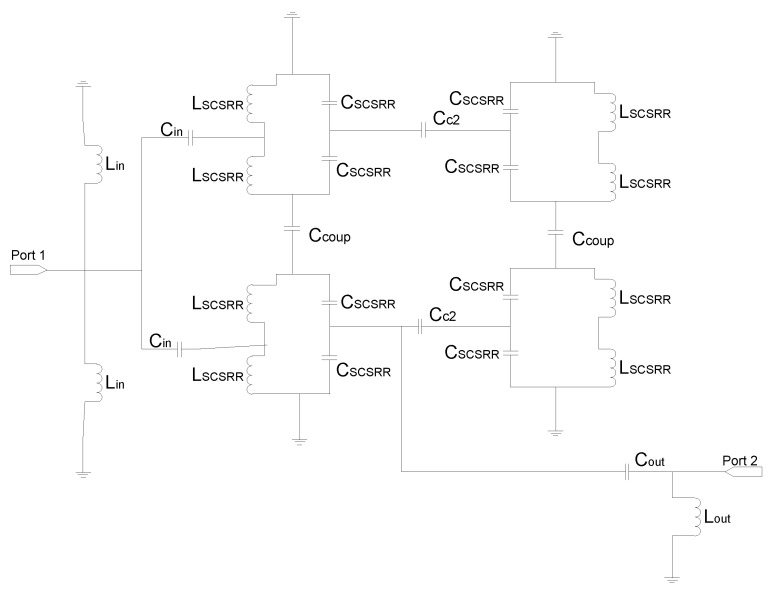
Equivalent circuit model of the two frequency resonators.

For the aforementioned configuration, the equivalent circuit is analyzed as follows: the input line finishes when it reaches the first SCSRR. The end of the line is simulated as an open stub. In Figure 16, an inductor *L_in_*. is placed as the open stub between the line and the SCSRR in the top ground plane, and a second one is placed from the line to the bottom SCSRR. Between the input line and the SCSRR’s top and bottom again, there is a coupler represented as *C_in_*. The parameters for the resonators are once again represented as *L_SCSRR_* and *C_SCSRR_*. The face-to-face configuration is achieved by facing each resonator on the coupler’s side. The coupling between resonators at the same level—that is, in the same metallization layer—is designed with the coupler *C_c2_*. The coupling between the rings of the different layers is characterized as *C_coup_*. As depicted in Figure 15, the output line starts again in the first resonator. Therefore, there is a coupler that starts in the first ring to the output, identified as *C_out_*. Finally, the output line below the resonator once again emulates as an open stub with the inductor *L_out_*.

**Figure 17:**
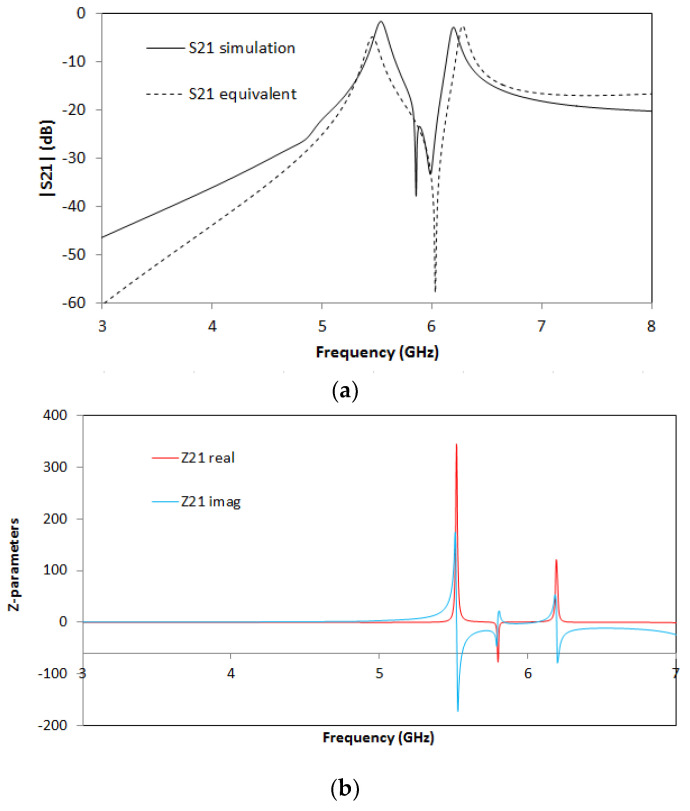
**(a**). Simulated S21 (solid black line), simulated S21 for the equivalent circuit model (dashed black line). (**b**) Impedance graph: Z21 real part (solid red line), Z21 imaginary part (solid blue line).

The dimensions of the SCSRRs used are the same for all of them, and they have the same dimensions as those in the circuit depicted in Figure 4. The dimensions that change in this case are the distance by which the input and output lines overlap the rings, defined in Figure 8 as *p_1_* and *p_2_*. In this case, *p_1_* = 0.7 mm and *p_2_* = 0.7 mm. Finally, a relevant parameter is introduced in this device, i.e., the separation between the two resonators in the same layer, identified as *sep*. In this circuit, *sep* = 0.2 mm. For the enumerated dimensions, the values of the equivalent circuit’s components are: *L_in_* = 1.595 nH and *C_in_* = 0.188 pF, and the values of the resonator components are: *L_SCSRR_* = 0.695 nH and *C_SCSRR_* = 0.851 pF. The coupling between the resonator on the same layer *C_c2_* = 0.366 pF, and the coupling between the resonators in different layers *C_coup_* = 1.145 pF. Finally, *C_out_* = 0.266 pF and *L_out_* = 0.648 nH. The results of the electromagnetic simulation, in comparison with the result of the proposed equivalent circuit model, are plotted in Figure 17.

In this device, two resonances are achieved. The first one is obtained at *f_0_* = 5.536 GHz, with a value of insertion loss of S21 = −1.56 dB. The second resonance occurs at *f_1_* = 6.192 GHz, with an insertion loss of S21 = −2.81 dB. The ratio between the resonant frequencies *f_1_*/*f_0_* is 1.118. As the resonances are very close to each other, it provides design flexibility to this device.

**Figure 18:**
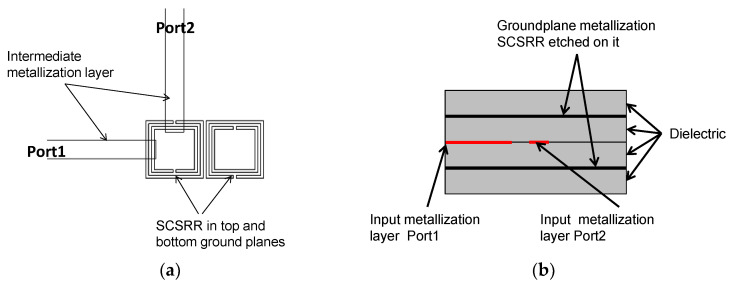
(**a**) Top view SCSRR multi-frequency side-by-side reverse resonator. (**b**) Front view of the stacked layer distribution.

On the second configuration, the side-by-side reverse configuration is used. The second main difference in this device is that the output port is also designed in stripline metallization. The top layout is displayed in Figure 18a. The input line is built on one side. It finishes just on the side of the SCSRRs. Two rings in reverse configuration are placed in both metallization layers. The output line is designed perpendicular to the input line, also in the same layer as the input line. The layer distribution layout is depicted in Figure 18b.

**Figure 19:**
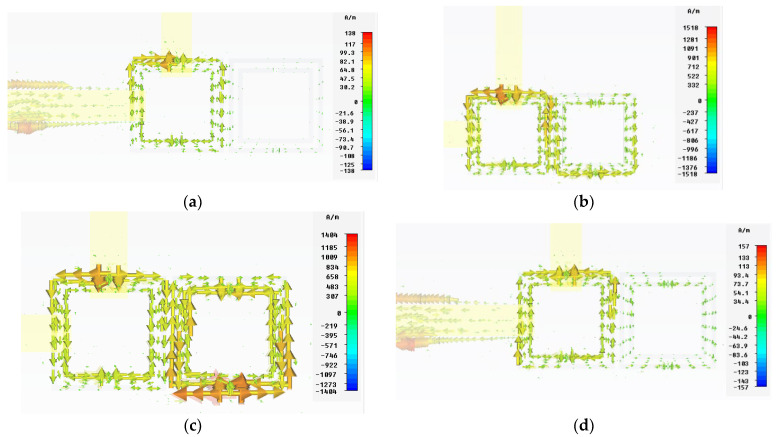
Vector surface current at resonant frequencies. (**a**) 5 GHz, (**b**) 5.82 GHz, (**c**) 6.32 GHz, (**d**) 7 GHz.

In Figure 19, it is shown how the surface current travels two different paths to the output at resonant frequencies. At 5.82 GHz, the currents flow through the perimeter of the first resonator and partly on the external ring of the second resonator. In the resonance at 6.32 GHz, the currents flow from the first resonator, they are coupled to the second, they flow through both the internal and external rings, and then they couple back to the first resonator to finally be coupled to the output line on port 2. In non-resonant frequencies, it can be seen that the SCSRRs are weakly excited; the currents flow through the ring to return to the input metallization strip (Figure 19a,d).

**Figure 20:**
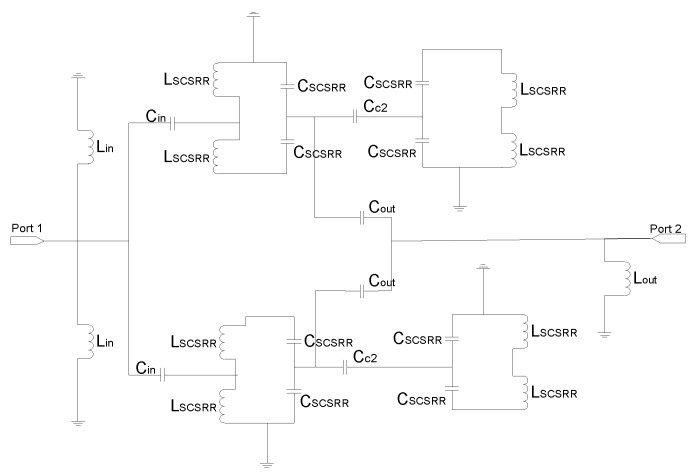
Equivalent circuit model of two frequency side-by-side reverse resonators.

The proposed circuit can be seen in Figure 20. The input line ends at the first resonator. The end of the line, as has been explained, behaves as an open stub from the line to the ground plane on the top layer, and it behaves the same to the bottom layer. This is emulated as the inductors *L_in_*. Again, the coupling between the input line and the top and bottom resonators is represented with capacitor *C_in_*. The resonators are represented with their lumped components *L_SCSRR_* and *C_SCSRR_*. To achieve the reverse position, in the circuit, the resonators are rotated vertically. As displayed in the top layout, the output line starts at the level of the first ring; thus, the top and bottom output lines are born in the first rings, and they are coupled to the output line with a coupler *C_out_*.

**Figure 21:**
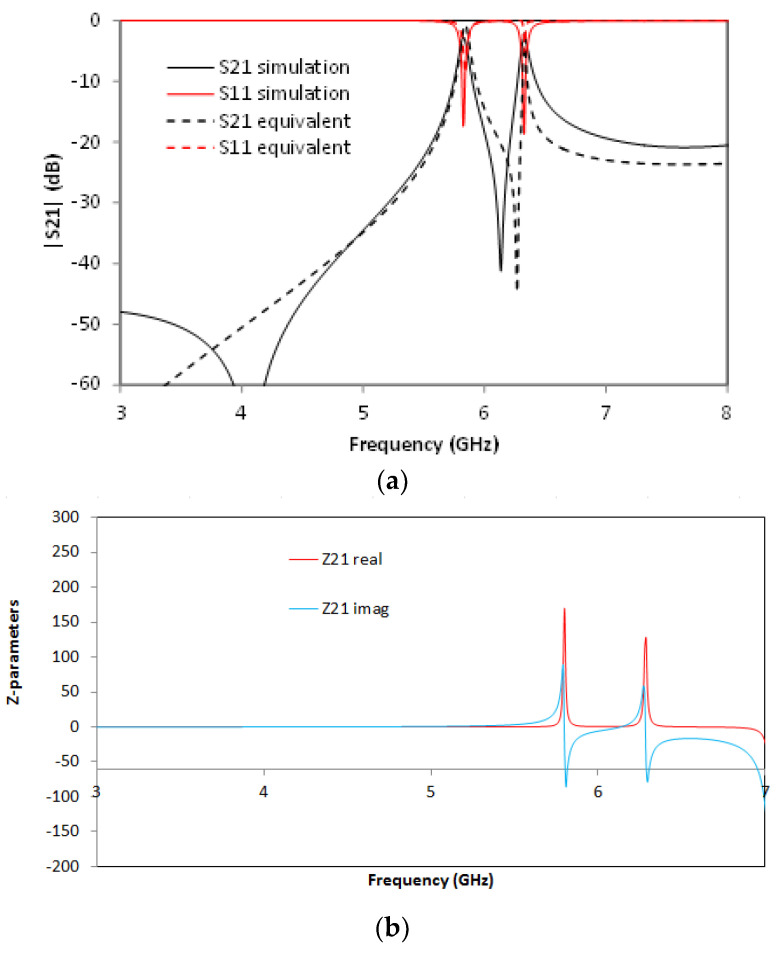
(**a**) Simulated S21 (solid black line), simulated S21 for the equivalent circuit model (dashed black line), simulated S11 (solid red line), simulated S11 for the equivalent circuit model (dashed red line). (**b**) Impedance graph: Z21 real part (solid red line), Z21 imaginary part (solid blue line).

The dimensions of the resonators are the same as those in previous devices. In this case, the separation between the resonators on the same layer is *sep* = 0.1 mm. The parameters *p_1_* and *p_2_* are, in this device, *p_1_* = 0.6 mm and *p_2_* = 0.8 mm. With all the provided dimensions for the device described, the values of the equivalent circuit provided are the following: *L_in_* = 0.995 nH, *C_in_* = 0.108 pF, *L_SCSRR_* = 0.745 nH, *C_SCSRR_* = 0.816 pF, coupling between resonators *C_c2_* = 0.106 pF, coupling between resonators to the output line *C_out_* = 0.108 pF. Finally, the open stub built in the output line is *L_out_* = 0.995 nH. In Figure 21, the S-parameters of the simulation results in comparison with the equivalent circuit model are presented.

The results exhibit two resonances: the first one at *f_0_* = 5.832 GHz, with a value of S21 = −2.87 dB, and the second one at *f_1_* = 6.328 GHz, with a value of S21 = −2.97 dB. From the figure, two results can be highlighted. First, the ratio between the resonant frequencies *f_1_*/*f_0_* is 1.085, which is even better than that for the previous device presented. Second, the equivalent circuit proposed matches the values of the simulated circuit with very good agreement. In the impedance graph of Figure 21b, the variation of the impedance at the resonant frequencies is achieved, once more caused by the impedance to capacitance.

## 6. Diplexers

**Figure 22:**
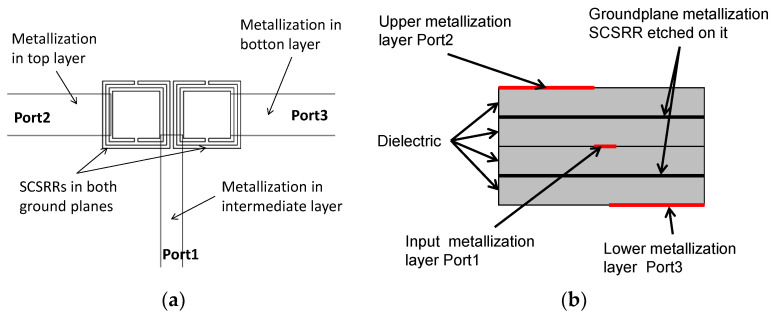
(**a**) Top view of the overlapped layer distribution of a two-layer duplexer. (**b**) Front view of the layer distribution.

In this section, a multilayer stripline diplexer is proposed. Diplexers using composite RH/LH media were designed in microstrip technology [40,41]. It is common to exploit the use of resonant particles to build diplexers, either using non-bianisotropic resonators [42], modified Split Ring Resonators [43], or magnetically coupled resonators [44]. The use of RH/LH media in the multi-layer stage was proposed by [45], obtaining a compact device. Analytical models for multi-stage resonator diplexers were already provided [46,47]. The following device exhibits some specific characteristics. In both of the ground plane metallization layers, two SCSRRs in a side-by-side configuration are placed. The stripline acts as a feed for the device, as shown in Figure 22a. The input layer reaches the resonators just in the middle of them. The output lines are microstrip lines on the top and bottom layers and are perpendicular to the input line. The layer distribution is depicted in Figure 22b.

**Figure 23:**
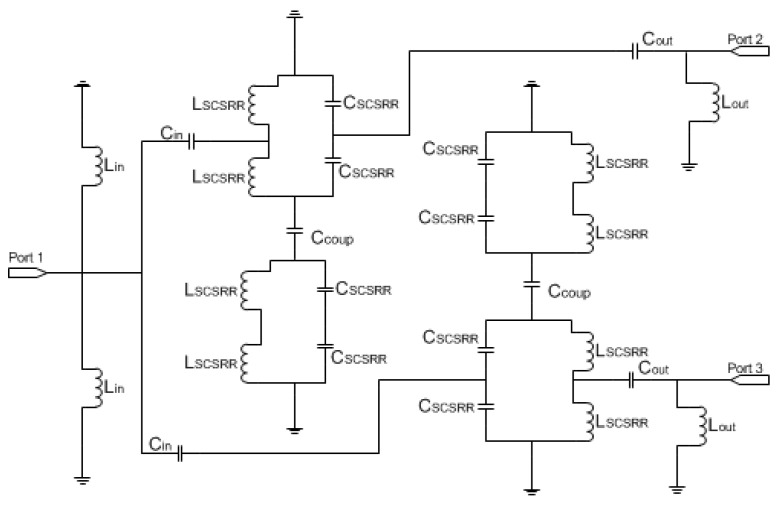
Equivalent circuit model for the multilayer stripline diplexer.

The equivalent circuit of this device explains the behavior of the circuit (see Figure 23). The end of the input stripline is emulated as an open stub characterized as an open stub *L_in_*, as already explained in this article. The input line is coupled to the top and bottom SCSRRs. This coupling is modeled with capacitor *C_in_*, with all the SCSRRs having the same dimensions. SCSRRs are modeled with LC tanks, and, in the figure, they are modeled in the equivalent circuit with serial couples of capacitors *C_SCSRR_* and inductors *L_SCSRR_*. It is particular for this device that the top resonator on the left side is only coupled to the resonator on the bottom layer just below it. This coupling is characterized with the capacitor *C_coup_*. It is worth noting that there is no coupling between the rings close to each other, a fact that has been demonstrated empirically in [48]. Finally, the SCSRR on the top is coupled with the output line on the top with the capacitor *C_out_*, and the end of the output line is modeled as an open stub painted as inductor *L_out_*, which is exactly the same approach as the line to port 3. The input stripline is coupled to the bottom-right SCSRR, modeled with the coupler *C_in_*. This resonator is coupled to the SCSRR just above it, coupling *C_coup_*, but not to the ring in the ground plane just on its left side. Finally, energy is coupled with the output microstrip line to port 3, modeled as a coupler *C_out_*, and the matching open stub of this output line is emulated with the inductor *L_out_*.

For the dimensions of the resonators of the rings detailed above, the following values of equivalent circuit lumped components are considered: input values—*L_in_* = 1.235 nH, *C_in_* = 0.188 pF; values for the resonators—*L_SCSRR_* = 0.695 nH, *C_SCSRR_* = 0.740 pF; coupling between resonators—*C_coup_* = 1.874 pF; output values—*C_out_* = 0.376 pF, *L_out_* = 0.496 nH.

**Figure 24:**
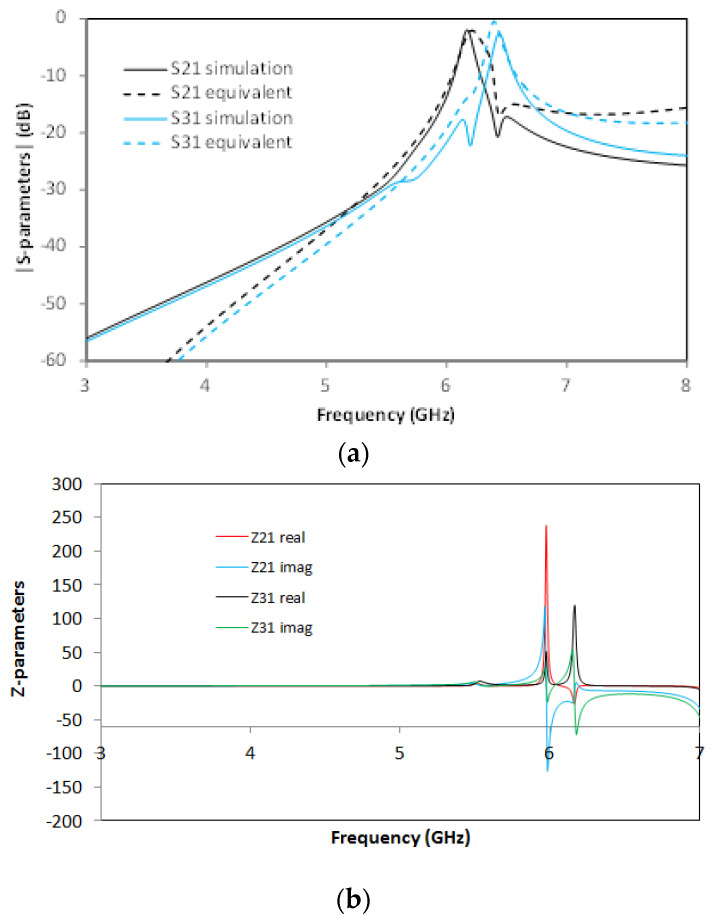
(**a**) Simulated S21 (solid black line), simulated S21 for the equivalent circuit model (dashed black line), simulated S31 (solid blue line), simulated S31 for the equivalent circuit model (dashed blue line). (**b**) Impedance graph: Z21 real part (solid red line), Z21 imaginary part (solid blue line), Z31 real part (solid black line), Z31 imaginary part (solid green line).

The output for this device shows two different resonant frequencies. The resonant frequency to port 2 is obtained at *f_0_* = 6.176 GHz with an insertion loss S21 = −2.02 dB, and the resonant frequency to port 3 is obtained at *f_1_* = 6.44 GHz with an insertion loss S31 = −2.56 dB. The remarkable result for this device is not the insertion losses on each port but that the output resonant frequencies are really close to each other on a symmetric device. The good agreement between the simulation and the equivalent circuit output validates the analysis described. As for the previous device, the impedance graph in Figure 24b shows the effect of the capacitor and the inductor of the resonator.

## 7. Filters

**Figure 25:**
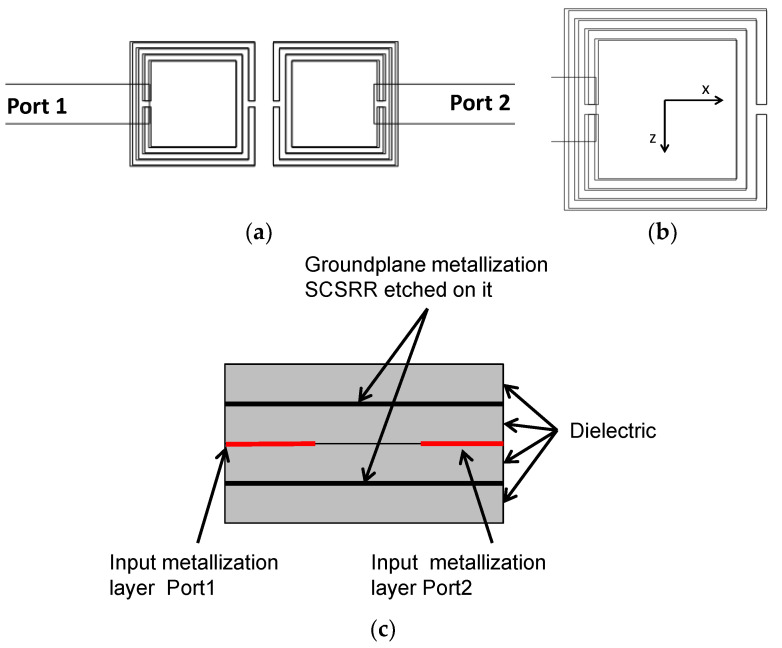
(**a**) Top view of the overlapped layer distribution of a multilayer stripline filter. (**b**) SCSRR zoom. (**c**) Front view of the layer distribution.

In the last section of this work, a device acting as a filter is described. The design of filters with SRR and CSRR in planar technologies has been widely studied [13,49]. The use of couples of CSRRs for building resonators was analyzed in detail for microstrip and coplanar transmission lines [50]. The use of pairs of CSRRs in different combinations was proposed in an SIW transmission line [32] to design filters, along with the use of stripline as a transmission line to build multi-layer filters [51]. For the design proposed in this work, the face-to-face layout is chosen. The input and output lines are placed in a serial configuration. Both are in the same metallization layer. The top layout is shown in Figure 25a. The main advantage in the device presented is that the second pair of CSRRs is placed just above the first one. The effective area needed to obtain similar results is reduced by more than 60%. The dimensions of the rings in the lower ground plane are the same as those in previous devices. In this case, the separation of SCSRRs is *sep* = 0.6 mm. The main difference in this case is that the dimensions of the rings on the top ground plane are scaled by a factor of 1.02 on both dimensions x and z. A zoom with a detailed view is provided in Figure 25b. The distance from the border of the first SCSRR to the end of the output line is *p_1_* = 0.55 mm. The distance from the end of the output line to the border of the SCSRR is defined as *p_2_* = 0.7 mm.

**Figure 26:**
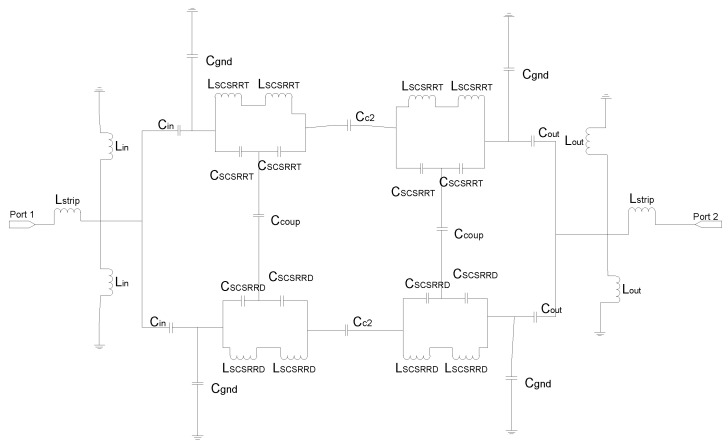
Equivalent circuit model for the multilayer stripline filter.

The equivalent circuit of the previous device is represented in Figure 26. The input line is represented with the per unit length inductance *L_strip_*. The input line is cut just when reaching the resonators. This is emulated, as previously stated, with open stubs: one to the top ring and another one to the bottom ring—both with the same value *L_in_*. The coupling between the input line and resonators is modelled by *C_in_*. In this circuit, as resonators on different layers have different dimensions, they have been named with different labels. The rings in the bottom layer are characterized with the LC tank, labeled *L_SCSRRD_* and *C_SCSRRD_*. The LC components for the resonators on the top layer are labeled as *L_SCSRRT_* and *C_SCSRRT_*. The coupling between the resonators and the ground plane is depicted as *C_gnd_*. The coupling between the resonators in the same layer as *C_c2_* and the last coupling effect are the coupling between the rings on different layers, which is characterized as *C_coup_*. The output components are pretty similar to the input ones. In this case, the coupling between the resonators and output line is modeled with *C_out_*. The distance by which the output line overlaps the rings is again emulated as open stubs, represented as *L_out_*, and, finally, the output line is represented as the input one, like *L_strip_*.

**Figure 27:**
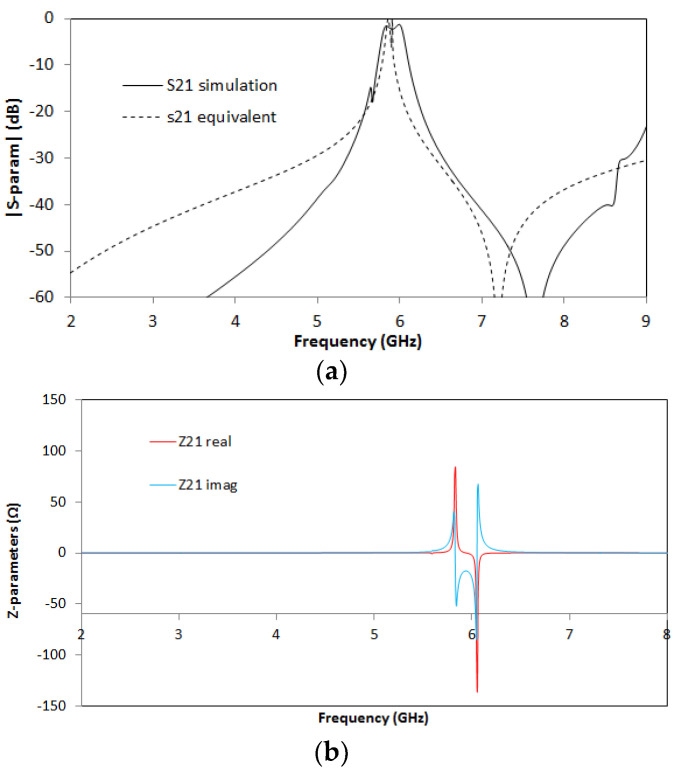
(**a**) Simulated S21 (solid black line), simulated S21 for the equivalent circuit model (dashed black line). (**b**) Impedance graph: Z21 real part (solid red line), Z21 imaginary part (solid blue line).

For the dimensions stated above, the values of the components in the equivalent circuit are: *L_strip_* = 0.895 nH, *L_in_* = 3.04 nH, *C_in_* = 0.138 pF, *C_gnd_* = 0.158 pF. The LC values of the resonator on the bottom layer *L_SCSRRD_* = 0.670 nH, which is half of the value of the resonator calculated in [22] and the capacitor *C_SCSRRD_* = 0.740 pF. In this case, the value of each capacitor in serial is double that of the single capacitor calculated in [22]. The values for the LC components in the top layer are *L_SCSRRT_* = 0.6753 nH and *C_SCSRRT_* = 0.7347 pF. The coupler between the resonators in the same layer *C_c2_* = 0.204 pF. The coupler between the resonators on different layers *C_coup_* = 1.38 pF, *C_out_* = 0.138 pF. The output inductor *L_out_* = 3.04 nH. The S-parameters for the simulation values of the circuit in comparison with the equivalent circuit are shown in Figure 27.

The results for the filter are promising. The insertion loss S21 = 1.01 dB, the central frequency *f_0_* = 5.920 GHz, and the BW at −3 dB is 270 MHz.

## 8. Summary

As a summary for this article, in Table 1, the characteristics of the different configurations presented in this work are presented.

In relation to the effective area calculation, the ring dimensions plus an additional distance of 3 mm on each side have been considered. In the filter device, the center of the passband is chosen to calculate λg. On the multi-frequency resonators and duplexer, with two resonant frequencies each, the higher frequency is chosen to provide the least favorable result. Finally, in Table 2, the differentiating results and the other device-specific parameters, such as the unloaded quality factor (Qu) for resonators, are enumerated.

## 9. Conclusions

Stripline transmission lines are suitable to excite Complementary Split Ring Resonators placed on the ground plane due to the dominant TEM propagating mode. Starting with this assumption, different devices in multilayer structures are presented. A high performance in miniaturized devices is obtained, such as in power dividers, duplexers resonators, and filters. As the resonators are designed in stacked configurations, the effective size of the device is decremented. The stacked configuration also provides flexibility in the design, as, in some of the proposed devices, the output line can be placed in different layers with the same response. The proposed devices therefore exhibit the following properties, which can aid in the design of more compact and flexible devices: (i) they employ a stacked configuration, taking advantage of a volumetric configuration instead of increasing the circuit footprint in a configuration that is amenable with chip housings; (ii) the structure is fully planar and compatible with planar transmission lines; (iii) the proposed devices can have outputs in different facets, enabling a more flexible routing of connections in transceiver systems. An analytical model is presented for each device, considering the effect of coupling between which different resonances excite. These, in turn, can be advantageously used to implement multi-frequency resonators and filters. As future work, measurement validations of the final design should be performed. The CSRR elements in different planar transmission line configurations have been validated in measurements (including multi-stack SIW-based configurations), showing a good agreement with the design parameters and simulation results. The current limitations within our lab in terms of probe connections and fixtures, as well as the potential expansion towards tunable frequency response devices aided by active circuit inclusion or the use of variable ε or µ substrates and materials (e.g., liquid crystal or ferromagnetic substrates), are future work lines to be developed.

## Figures and Tables

**Table 1:** Summary of the relevant parameters.

Device	f_0_ – f_1_ (GHz)	Effective Area λ_g_^2^	Insertion Loss (dB)	Return Loss (dB)	f_1_/f_0_ Ratio
**[Power divider—** **Figure 7** **]**	6.008	0.09	3.43/3.67	36.2	N.A.
**[Resonator—** **Figure 11** **]**	6.048	0.09	1.01	18.25	N.A.
**[Multi-frequency—** **Figure 15** **]**	5.536/6.192	0.129	1.56/2.81	8.35/6.88	1.118
**[Multi-frequency—** **Figure 18** **]**	5.832/6.328	0.139	2.87/2.97	17.31/18.54	1.085
**[Duplexer—** **Figure 22** **]**	6.176/6.440	0.144	2.02/2.56	8.42/6.54	1.043
**[Filter—** **Figure 25** **]**	5.920	0.125	1.01	5.01	N.A.

**Table 2:** Summary of the relevant parameters.

Device	Highlights
**[Power divider—** **Figure 7** **]**	Efficiency: 93%
**[Resonator—** **Figure 11** **]**	Q_u_: 355
**[Multi-frequency—** **Figure 15** **]**	Q_u1_: 448 Q_u2_: 302 Ratio between frequencies: 1.118
**[Multi-frequency—** **Figure 18** **]**	Q_u1_: 273 Q_u2_: 267 Ratio between frequencies: 1.085
**[Duplexer—** **Figure 22** **]**	Ratio between frequencies: 1.044
**[Filter—** **Figure 25** **]**	Effective area: 0.125

## Data Availability

Not applicable.
